# Objective response to mTOR inhibition in a metastatic collision tumor of the liver composed of melanoma and adenocarcinoma with *TSC1* loss: a case report

**DOI:** 10.1186/s12885-017-3167-y

**Published:** 2017-03-16

**Authors:** Munveer S. Bhangoo, Jenny Y. Zhou, Siraj M. Ali, Russell Madison, Alexa B. Schrock, Carrie Costantini

**Affiliations:** 1 Division of Hematology Oncology, Scripps Clinic, 10666 N. Torrey Pines Ave, La Jolla, CA 92037 USA; 2 Department of Internal Medicine, Scripps Mercy Hospital, San Diego, CA USA; 3 Foundation Medicine, Cambridge, MA USA

**Keywords:** Collision tumor, Uveal melanoma, *TSC1* mutation, mTOR inhibition, Temsirolimus, Carcinoma Undetermined Primary, Melanoma, Next Generation Sequencing, Case Report

## Abstract

**Background:**

Collision tumors are uncommon but well described clinical entities composed of distinct tumor histologies occurring within the same anatomic site. Optimal management of patients with collision tumors remains highly variable and depends on clinical characteristics such as the involved tumor types, predominant histology, as well as the extent of disease. Comprehensive genomic profiling is a means of identifying genomic alterations to suggest benefit from targeted therapy.

**Case presentation:**

A 78-year-old woman presented to medical oncology with liver metastases occurring within the background of a 1-year history of uveal melanoma. Biopsy of the liver metastases revealed presence of adenocarcinoma along with nests of malignant melanoma consistent with a collision tumor. The disease was refractory to several lines of conventional cytotoxic chemotherapy, and the patient later developed pulmonary metastases while on chemotherapy. The patient’s tumor tissue was assayed by comprehensive genomic profiling which revealed presence of a *TSC1* partial loss. The patient was subsequently initiated on temsirolimus 15 mg intravenously weekly for 4 months. Restaging imaging demonstrated a partial response to therapy by RECIST 1.1 criteria and clinical benefit for 6 months until the patient passed away secondary to unrelated causes.

**Conclusions:**

We report the first case of a collision tumor composed of adenocarcinoma and melanoma with a *TSC1* mutation that objectively and durably responded to mTOR inhibition.

## Background

Cancer therapy continues to move towards the targeting of molecular signaling pathways; mTOR inhibitors have been well studied since the early 1990s with increasing recognition immunosuppressive and anticancer properties. In particular, the *TSC1-TSC2* complex has emerged as an integral signal involved in the inhibition of mTORC1. Inactivating alterations of tumor suppressor genes *TSC1* and *TSC2* have been implicated in tuberous sclerosis and a wide variety of malignancies in which mTORC1 was found to be highly activated [1]. Promising clinical trials have shown that tumors harboring *TSC1* mutations respond to mTOR inhibitors and the clinical significance of such a mutation is highlighted in the present case [1].

We present a patient with a prior diagnosis of uveal melanoma with new metastatic hepatic disease for whom biopsy unexpectedly demonstrated a collision tumor of melanoma and adenocarcinoma of unknown primary. Although collision tumors have been described in literature, primarily between two cutaneous malignancies, a combination of melanoma and adenocarcinoma is extremely rare with only a few reported cases [2–5]. The patient did not respond to several conventional chemotherapy agents but did respond to targeted mTOR inhibition after genomic sequencing revealed the presence of a *TSC1* mutation. This case highlights two unique facets in both the phenomenon of collision tumors and the significance of targeted signaling pathway agents where traditional chemotherapy has failed.

## Case presentation

A 78-year-old woman presented to our institution for further management of newly diagnosed metastatic liver disease.

The patient initially presented with symptoms of decreased visual acuity of the right eye 1 year prior. Comprehensive physical examination was unrevealing. The patient was referred to ophthalmology and was diagnosed with choroidal melanoma. The tumor initially measured 19.5 x 13.6 mm and involved 50% of the optic nerve head as well as the macula. The patient was treated with radiotherapy along with transpupillary thermotherapy.

**Fig. 1:**
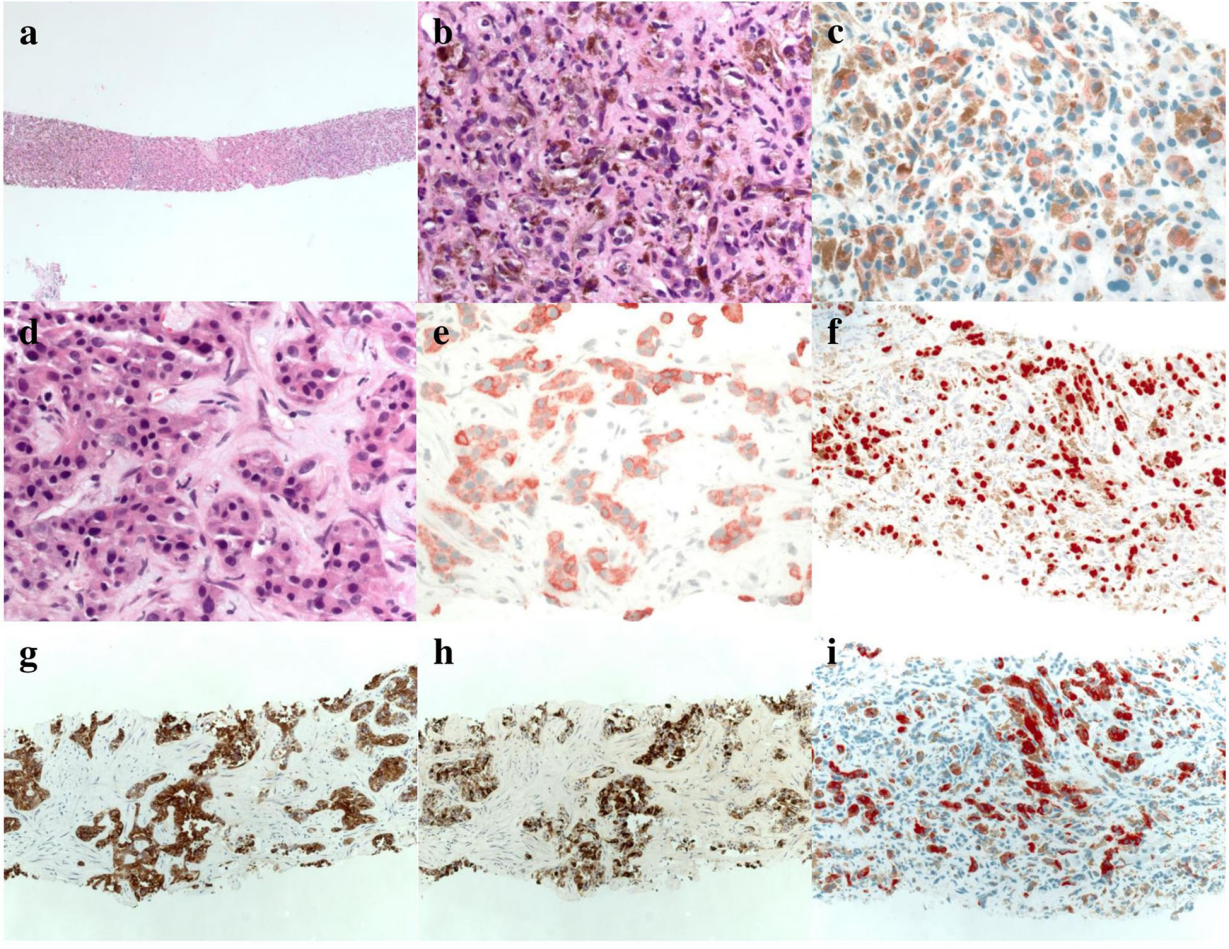
Core-needle liver biopsy. **a** low-powered field showing melanoma (*left*) and adenocarcinoma (*right*). **b** 40x view of melanoma. **c** S-100 stain. **d** 40x of carcinoma. **e** Pankeratin stain. **f** SOX10 stain. **g** CK7 stain. **h** CK20 stain. **i** HMB-45 stain

As part of routine surveillance for the diagnosis of uveal melanoma, the patient was followed with serial CT scans of the abdomen and pelvis every 3 months. Nine months after the initial diagnosis repeat CT scan revealed multiple liver masses suggestive of metastatic disease involving the lateral segment of the left hepatic lobe. A dominant mass was identified measuring 6.9 x 5.8 cm and the patient was referred for core needle biopsy. Pathology revealed abundant involvement by adenocarcinoma, which stained positive for pankeratin (AE1/AE3), CK7, CK20 (Fig. 1). Additionally, several nests of atypical cells with cytoplasmic pigmentation consistent with malignant melanoma were identified. Immunohistochemical stains showed the malignant pigmented neoplasm to be negative for AE1/AE3 and positive for S100, SOX10, HMB45 (Fig. 1).

Given the unexpected findings of metastatic adenocarcinoma co-existing with metastatic melanoma, a diagnostic workup was done to identify the site of origin for the adenocarcinoma component. Whole-body PET/CT, and upper and lower endoscopy failed to identify the primary anatomic site. The patient was therefore diagnosed with metastatic collision tumor composed of adenocarcinoma of unknown primary along with malignant melanoma and empiric chemotherapy was initiated. Unfortunately, the disease proved refractory to several lines of conventional cytotoxic chemotherapy. She received two cycles of oral capecitabine (Xeloda, Genentech, South San Francisco, CA; 1000 mg/m2 orally day 1–14 every 21 days). This was discontinued secondary to grade 2–3 gastrointestinal toxicity (nausea, vomiting). The patient subsequently received 8 cycles of gemcitabine (Gemzar, Eli Lilly, Indianapolis, IN; 900 mg/m2 IV day 1, 8 every 21 days;) combined with protein-bound nab-paclitaxel (Abraxane, Celgene, Summit, NJ; 100 mg/m2 IV day 1, 8, 15 every 28 days). Restaging imaging revealed progressive disease in the liver as well as the interval development of metastatic lung nodules.

The patient’s tissue was submitted as a formalin-fixed, paraffin-embedded block to a CLIA-certified, CAP-accredited laboratory (Foundation Medicine, Cambridge, MA) for CGP. DNA was extracted from the tumor specimen. Hybrid-capture-based CGP using next-generation sequencing was performed of the entire coding sequence. This included 236 genes and 47 introns of 19 genes involved in fusions at a depth of X500. Alterations were identified as point mutations, deletions, amplifications, duplications, insertions, rearrangements, and splice variants. These were characterized as known or likely pathogenic changes as reported by the FoundationOne assay.

DNA extracted from the biopsy of the collision tumor submitted for NGS contained both melanoma and adenocarcinoma. Several genomic alterations were identified as follows *TSC1* loss (homozygous deletion) of exons 20-23, *CDKN2A/B* loss, *BAP1* (E20fs*52), *PBRM1* (R1095fs*39). CGP is a validated approach in detecting base substitutions, short insertions, deletions, copy number alterations and selected fusion products. The technique has been directly compared to established assays including PCR. Test sensitivity approximately 95–99% across various alteration types with high specificity (positive predictive value >99%). Because CGP is a validated technique to identify genetic abnormalities, no additional testing on the tumor sample was clinically indicated [6].

Tuberous sclerosis gene 1 (*TSC1*) is a tumor suppressor gene found on the long arm of chromosome 9. *TSC1* encodes the protein gene product hamartin composed of one transmembrane domain and two coiled-coil domains. The first coiled-coil domain modulates interaction between hamartin and tuberin. A second coiled-coil domain is encoded by exons 17 to 23 which stabilizes the tuberin-hamartin complex [7]. The hamartin-tuberin complex enables the GTPase-activating function of tuberin and is a major regulator of small G-protein Rheb which interacts with mTORC1 [8, 9]. Loss of hamartin, therefore results in increased downstream mTOR activity potentially predicting increased sensitivity to mTOR inhibitors.

**Fig. 2:**
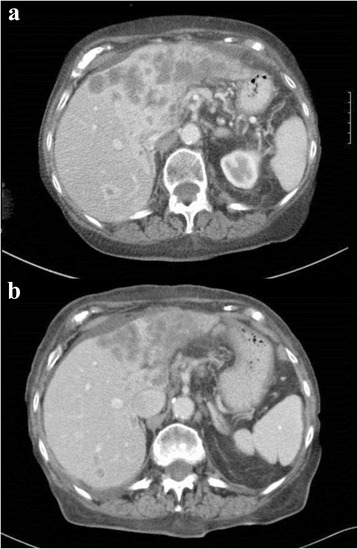
Pre-treatment and post-treatment imaging. **a** Pre-treatment CT scan of liver. **b** Post-treatment CT scan of liver

Based on the results of CGP the patient began treatment with temsirolimus (Torisel, Pfizer, New York, NY) 15 mg intravenously weekly. The patient was followed with clinical examinations monthly and restaging imaging every 2 months while on therapy. After 4 months of treatment, a restaging CT scan of the chest, abdomen, and pelvis demonstrated a significant partial response to therapy by RECIST 1.1 criteria (Fig. 2). Restaging imaging 2 months later demonstrated stable disease. The patient experienced no dose-limiting toxicities while on therapy. Unfortunately, the patient later developed severe sepsis related to *Clostridium Difficile* colitis and died from infectious complications 6 months after starting temsirolimus. Informed consent was obtained for the publication of this manuscript and available to the journal editors for review. This manuscript adhered to CARE guidelines for case reports.

## Discussion

A collision tumor is an uncommon occurrence in which two distinct tumor histologies are present in a single anatomic site. Under the World Health Organization (WHO) histological classification guidelines, a collision tumor should comprise at least two different malignant components with no mixed or transitional area between [10, 11]. Several theories have been proposed to explain the pathophysiology of this phenomenon. One mechanism involves the alteration of the tumor microenvironment facilitating colonization or metastases of tumor to that site. An alternative model would be a coincidental meeting of two primary tumors. In contra-distinction, a composite tumor references a tumor that evolves into two distinct histologies from presumably a pluripotent precursor cancer stem cell.

Although cases of synchronous adenocarcinoma and melanoma have been reported, the presentation remains exceedingly rare [2–5]. The clinical behavior and natural history of collision tumors may reflect the biology of the more aggressive tumor involved [11]. In general, both metastatic adenocarcinoma of undetermined primary and metastatic ocular melanoma are associated with poor prognoses. It is unknown whether or not the presence of a collision tumor portends a worse prognosis than either condition alone.

Mutations in *TSC1* and *TSC2* have been reported in a variety of neoplasms and benign tumors including pulmonary lymphangioleiomyomatosis (LAM), perivascular epitheloid cell tumors (PEComa), urothelial carcinomas, renal cell carcinoma and hepatocellular carcinomas [12]. Mutations in the tuberous sclerosis complex (TSC) has been reported in up to 14.5% of bladder cancer and 28.6% of hepatocellular carcinoma [13, 14]. Alterations resulting in *PTEN* loss and *PIK3CA* amplification*,* which are also targets for mTOR/P13K inhibitors, have been implicated in 7% and 9% respectively, in carcinomas of unknown primary [15]. Of greater clinical relevance, the presence of mutations involving *TSC1* or *TSC2* may predict a response to downstream inhibition of the mTOR pathway [1, 7].

This patient’s disease had been highly refractory to conventional chemotherapeutic agents, but did demonstrate sensitivity to temsirolimus. In addition, the patient’s CGP demonstrated mutation of BRCA1-associated protein 1 (*BAP1)* gene, which is frequently inactivated in metastatic uveal melanomas [16]. *BAP1* encodes a nuclear ubiquitin carboxyterminal hydrolase, which is a class of deubiquitinating enzymes, and contains binding domains for BRCA1 and BARD1 to form a tumor suppressor complex among other functions [16]. In uveal melanomas, somatic inactivating mutations of *BAP1* have been highly associated with onset of metastatic behavior thus suggesting a potential novel target for therapy [16].

Additionally, noted on NGS were alterations in CDKN2A/2B as well as PBRM1. CDKN2A/2B encode tumor suppressor gene products cyclin-dependent kinase 4 inhibitor A/B. This gene encodes a cycle-dependent kinase inhibitor that prevents activation of the CDK kinases thereby inhibiting cell cycle G1 progression [17, 18]. Interestingly, germ line mutations in CDKN2A have been associated with familial melanoma syndromes and may have reflected genetic alterations from this patient’s uveal melanoma. PBRM1 encodes Polybromo-1 (BAF180) which is a subunit of ATP-dependent chromatin-remodeling complex. It has an additional role as a cofactor in transactivation of nuclear hormone receptors [19]. This abnormality is most closely associated with clear cell renal cell carcinoma occurring in up to 30% of cases. In addition, it has been reported in up to 2–4% of colorectal adenocarcinoma [20, 21]. Unfortunately, no FDA approved therapies targeting CDKN2A/B or PBRM1 are currently available. If additional targeted agents had been available, combination therapy may have been an important treatment consideration in management of this patient’s disease.

In our patient, disease progression on multiple lines of cytotoxic chemotherapy suggests that targeted inhibition of mTOR pathway was critical in inducing tumor response. Because tissue composed of both adenocarcinoma and melanoma was submitted for NGS, it is unknown whether the genetic alterations observed were unique to either tumor histology. Given the radiographic response to therapy with mTOR inhibitor, no repeat biopsy was clinically indicated. Nonetheless, the potential of detecting significant therapeutic targets implies that patients with refractory collision tumors would benefit from genomic profiling.

## Conclusion

To our knowledge, this is the first case of a collision tumor composed of adenocarcinoma and melanoma with a mutation of the *TSC1* gene locus. The objective clinical response achieved with targeted inhibition of the mTOR pathway further highlights the clinical significance of the genetic alteration identified by NGS. As a result, NGS may have a potential utility in identifying actionable targets in collision tumors failing to response to traditional cytotoxic chemotherapy. Further investigation into the identification of driver genetic mutations in collision tumors and potential therapeutic targets is warranted.

## Abbreviations

BAP1: BRCA1 associated protein 1
BARD1: BRCA1 Associated RING Domain 1
BRCA1: Breast cancer type 1 susceptibility protein
CDKN2A/B: Cyclin-dependent kinase inhibitor 2A/B
CGP: Comprehensive genomic profiling
CK: Cytokeratin
CLIA: Clinical Laboratory Improvement Act
CT: Computerized tomography
GNAQ: G protein subunit alpha q
GNAS: G protein subunit alpha S
GTPase: Guanosine-5’-triphosphate hydrolase
HMB45: Human Melanoma Black common marker to confirm melanoma
mTOR: Mammalian target of rapamycin
P13K: Phosphatidylinositide 3-kinases
PET/CT: Positron emission tomography computerized tomography
PIK3CA: Phosphatidylinositol-4, 5-bisphosphate 3-kinase catalytic subunit alpha
PRM1: Protein polybromo-1
PTEN: Phosphatase and tensin homolog
RECIST: Response Evaluation Criteria in Solid Tumors
S100 protein: Common marker for neural tissues and melanoma
SOX: Sry-related HMG box
TSC: Tuberous sclerosis
